# Total Antioxidant Capacity and Lipid Peroxidation in Semen of Patient with Hyperviscosity

**DOI:** 10.22074/cellj.2015.500

**Published:** 2015-01-13

**Authors:** Issa Layali, Eisa Tahmasbpour, Manijeh Joulaei, Seyed Gholam Ali Jorsaraei, Parvin Farzanegi

**Affiliations:** 1 Department of Biochemistry, Islamic Azad University, Sari Branch, Sari, Iran; 2 Member of Young Research Club, Islamic Azad University, Sari Branch, Sari, Iran; 3 Department of Psychology, Islamic Azad University, Sari Branch, Sari, Iran; 4 Fateme Zahra Infertility and Health Reproductive Research Center, Babol University of Medical Sciences, Babol, Iran; 5 Department of Exercise Physiology, Islamic Azad University, Sari Branch, Sari, Iran

**Keywords:** Male Infertility, Antioxidants, Lipid Peroxidation, MDA

## Abstract

Semen hyperviscosity (SHV) is one of the factors involved in deficiency in sperm function. This research aimed to evaluate seminal plasma total antioxidant capacity (TAC) and malondialdehyde (MDA) levels in infertile patients with hyperviscous and non-hyperviscous semen samples to understand whether hyperviscous semen is associated with oxidative damage in infertile subjects. In this cross sectional study, 59 semen samples were provided by fertile (n=12) individuals as control, infertile patients with normal viscosity (n=25) and infertile patients with hyperviscosity (n=22). After semen parameters examination, semen viscosity was studied by glass pipettes. Seminal plasma TAC and MDA levels were measured by ferric reducing of antioxidant power (FRAP) and thiobarbituric acid reaction (TBAR) methods, respectively. A probability less than 0.05 was considered statistically significant throughout the article. The mean of sperm parameters including: counts, motility and normal morphology in patients with hyperviscosity were significantly lower than those in non-hyperviscosity patients (p<0.05, p<0.01 and p<0.001, respectively). The mean of seminal plasma TAC value in seminal plasma of non-hyperviscosity patients (1710.31 ± 458.67 µmol/l) was significantly (p<0.01) higher than that of hyperviscosity group (1230.25 ± 352 µmol/l). A trend toward a higher mean of seminal plasma MDA value was estimated for hyperviscous group compared with non-hyperviscous (1.01 ± 0.41 nmol/ml vs. 0.94 ± 0.28 nmol/l); however, it was nonsignificant. Hyperviscous semen impairs seminal plasma TAC which is eventually associated with sperm membrane lipid peroxidation.

Sperm deficiency induced by oxidative stress (OX) is one of the main idiopathic factors that affect male fertility (1, 2). It occurs as a result of an imbalance between the productions of reactive oxygen species (ROS) and the available antioxidants defense system. Human sperm cells during the aerobic metabolism produce different types of ROS, which are highly reactive oxidizing agents and belong to the class of free radicals (2-4). Excessive ROS can be produced by immature sperm and leukocyte cells as well (5). Although they play a significant role in many biological processes, in high levels, they can impair normal sperm function by peroxidation of unsaturated fatty acids in membrane of spermatozoa and by DNA fragmentation (5, 6). They attack the fluidity of sperm plasma membrane, with subsequent loss of the ability for oocyte fusion and fertilization (7-9). In order to counteract the toxic effects of ROS, human semen includes several enzymatic antioxidants such as superoxide dismutase (SOD); glutathione peroxidase/ reductase system (GPX) and catalase (CAT); as well as non-enzymatic antioxidants such as vitamin C, E, Zn, Cu and glutathione that are referred as total antioxidant capacity (TAC) (2). This antioxidant system also compensates for the loss of sperm cytoplasmic enzymes, particularly SOD, CAT and vitamin C during the sperm development and maturation (2, 10). Changes in levels of oxidative damage factors in semen and their relationship to seminal fluid viscosity are not well known. Semen hyperviscosity (SHV) or delayed liquefaction is a condition that can seriously impair the physical and chemical characteristics of seminal fluid (11). It is characterized by a thick and congealed appearance; however, the coagulation and liquefaction of semen and its physiological characteristics is still not fully understood. It seems to be associated with reduced sperm motility, possibly due to a 'trapping effect', that prevents normal sperm progression through the female genital tract (7, 11-13). Although the negative effects of hyperviscosity on sperm parameters quality, especially on motility, are well known, the mechanism in which hyperviscous semen affecting spermatozoa are poorly understood. It appears that one of these mechanisms includes seminal antioxidants depletion and high sperm membrane lipid peroxidation, showing to have negative effects on sperm quality and function. Therefore, the general aim of this study was to determine whether the hyperviscous semen is associated with seminal plasma TAC depletion and sperm membrane lipid peroxidation in infertile patients.

In this cross sectional study, 59 semen samples were collected from fertile (n=12) individuals as control, infertile patients with hyperviscosity (n=22), infertile patients without hyperviscosity (n=25) who were referred to the Fatemeh Zahra *In Vitro* Fertilization (IVF) Center, Babol, Iran. An informed consent form was obtained from all participants prior to any involvement. Semen samples were obtained by masturbation into a sterile container after sexual abstinence for 2-3 days. Before semen analysis, a questionnaire was distributed to obtain information on age and lifestyle including: smoking habits, alcohol use, use or abuse of other substances and drugs, history of orchitis, testicular trauma, sexually transmitted disease, varicocele, inguinal hernia operation, cryptorchism, etc.

After collection, semen specimens were allowed to liquefy at room temperature for 30 minutes, and then used for analysis. A single sample provided by each subject was examined according to the World Health Organization (WHO) criteria, which is summarized in our previous study and analyzed for the appearance, volume and consistency (14). On microscopic examination, sperm concentration, percentage of normal morphology and motile sperm were objectively evaluated. Sperm count and motility were measured according to WHO criteria, whereas the percentage of sperm morphology was performed using eosin stain method according to Kruger’s strict criteria (15). Semen consistency was estimated by introducing a glass rod into the sample and measuring the length of the thread forming on withdrawal of the rod. Ejaculates with normal consistency had a thread length <2 cm, whereas semen classified as hyperviscous showed a thread length >2 cm (13).

Five hundred μl of semen sample were centrifuged at 1400 xg for 7 minutes at 4˚C. The supernatants were removed from pellets and diluted 10-fold with distilled water (add 900 μl distilled water to 10 μl seminal plasma), then immediately used for TAC assay. TAC was measured by ferric reducing of antioxidant power (FRAP) method described by Benize (16). Briefly, 1.5 ml of FRAP reagent, including 300 mM acetate buffer (pH=3.6), 10 mM 2,4,6- Tri (2-pyridinyl)-S-triazine (TPTZ) (Sigma Aldrich), and 20 mM ferric chloride, was added to each tube and kept in water bath at 37˚C for 5 minutes. Then, 50 μl of diluted seminal plasma was added to each tube, and kept again in water bath at 37˚C for 10 minutes. Eventually, the absorbance values of blank, standards (125, 250, 500 and 1000 μM/l FeSO_4_) and samples were estimated by spectrophotometer at 593 nm.

Seminal malondialdehyde (MDA) levels were analyzed according to methods described by Rao et al. (17). MDA was assessed using the thiobarbituric acid reaction (TBAR) method. Briefly, semen samples were centrifuged for 7 minutes at 2000 xg, and then 100 μl of seminal plasma (supernatants) was added in 900 μl of distilled water into a glass tube. To each tube, 500 μl of thiobarbituric acid reagent, including 0.67 g of 2-thiobarbituric acid dissolved in 100 ml of distilled water with 0.5 g NaOH and 100 ml glacial acetic acid, was added, and then heated for 1 hour in a boiling water bath. After cooling to room temperature, each tube was centrifuged for 10 minutes at 4000 xg, and the supernatant absorbance was read on a spectrophotometer at 534 nm.

All data are reported as means ± standard deviation (SD). An independent t test was considered to compare the scores of each of the measures and some of the parameters between the two groups. ANOVA model was utilized for statistical analyses of TAC and MDA concentrations among all groups. A probability less than 0.05 was considered statistically significant. Data were analyzed using the statistical package for the social sciences (SPSS) (SPSS Inc., Chicago, IL, USA) version 16.

The mean values of examined sperm parameters in the fertile and infertile men are shown in table 1. There were nonsignificant differences among all groups regarding mean values of semen volume and age. The mean age values of fertile, infertile patients with hyperviscous and non-hyperviscous were 31.21 ± 4.07, 29 ± 3.61 and 29 ± 3.37 years, respectively. Sperm count, sperm motility and sperm normal morphology in fertile group were significantly (p<0.001, p<0.001 and p<0.01, respectively) higher than those in both infertile groups (Table 1). In addition, the mean values of sperm parameters, including count, motility and normal morphology, in patient with hyperviscosity were significantly lower than those values in non-hyperviscosity patients (p<0.05, p<0.01 and p<0.001, respectively).

Comparison of TAC and MDA levels in the seminal plasma groups are shown in figures 1 and 2. The mean concentration of seminal TAC among groups was significantly different (p<0.001, Fig 1). The mean value of TAC in seminal plasma of fertile men (2346 ± 743.54 μmol/l) was considerably higher than the related values in patients with hyperviscous (1230.25 ± 352 μmol/l) and non-hyperviscous (1710.31 ± 458.67 μmol/l) semen (p<0.001). Moreover the mean value of TAC level in hyperviscous group was significantly lower than the related value in non-viscous group (p<0.01).

The mean of seminal MDA levels among groups was significantly different (p<0.01, Fig 2). The mean value of MDA in seminal plasma of fertile men (0.62 ± 0.18 nmol/ml) was significantly lower than the related values in patients with hyperviscous (1.01 ± 0.41 nmol/ml) and non-hyperviscous (0.94 ± 0.28 nmol/l) semen (p<0.001). A trend toward a higher mean of MDA value was seen for hyperviscous compared with non-hyperviscous; however, this difference was not significant (Fig 2).

**Table 1:** Sperm parameters quality in men according to viscosity


Sperm parameters	Fertile mennon-viscous	Infertile men withhyperviscous semen	Infertile men withnon-hyperviscous semen	P value

**Semen volume (ml)**	4.32 ± 1.12	3.25 ± 1.37	4 ± 1.41	0.12
**Sperm count (×10^6^/ml)**	87.53± 13.26	29.32 ± 25.35	46.8± 26.29	<0.001
**Total sperm count (×10^6^)**	394.36 ± 143.24	99.23 ± 99.29	189.9 ± 115.62	<0.001
**Sperm motility (%)**	68.39± 8.72	30.95 ± 19.11	52.8± 15.41	<0.01
Sperm normal morphology (%)*	15.68± 3.85	4.23 ± 2.5	7.56± 3.16	<0.001


Results are presented as mean ± SD and *; According to Kruger’s criteria.

**Fig 1:**
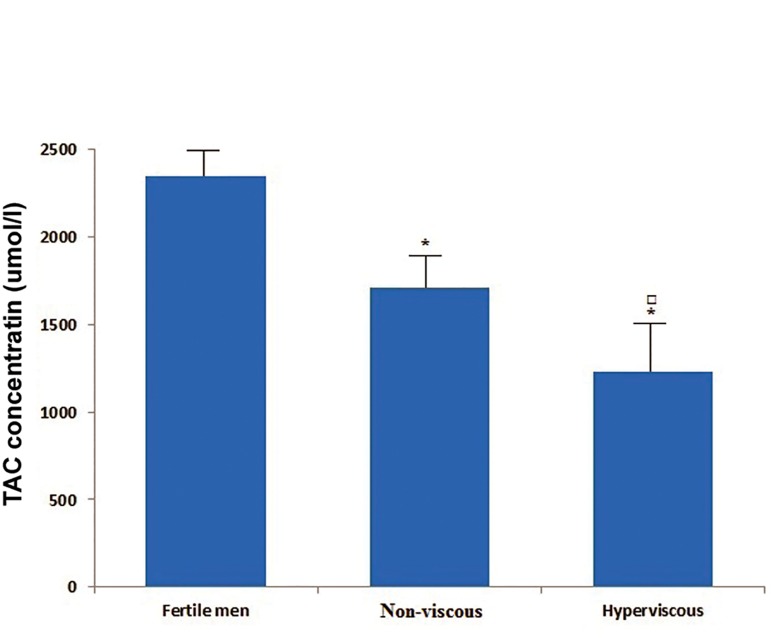
Comparison of mean (SD) values of seminal plasma TAC levels among groups. *; P<0.001 with respect to fertile group, ם; P<0.01 with respect to non-viscous group and TAC; Total antioxidant capacity.

**Fig 2:**
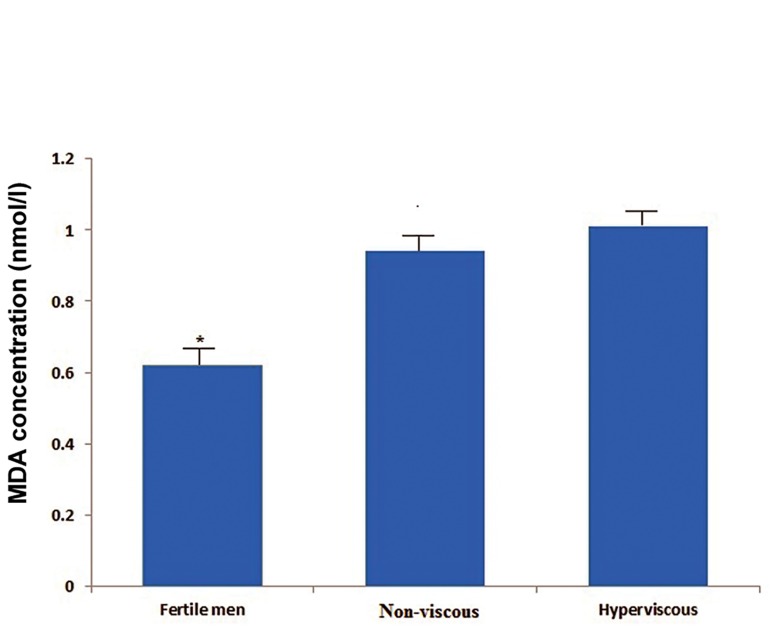
Comparison of mean (SD) values of seminal plasma MDA levels among groups. *; P<0.001 with respect to infertile patients with non-viscous and hyperviscous semen and Malondialdehyde (MDA).

Hyperviscosity is always associated with male infertility since the spermatozoa are tangled in the fibrous or mucoid mass in the semen and subsequently prevent the movement of sperm through the female genital tract. Also it causes difficulties in assisted reproductive technique (ART) because hyperviscous semen is always difficult to be manipulated *in vitro* and also considered hard to obtain the spermatozoa of good quality (11). Hyperviscosity of liquefied semen is a biophysical alteration of an ejaculate whose biochemical etiology is scarcely known, despite the different studies that have been carried out on this topic (11-13, 18-22). It seems not to be due to a single pathogenic factor, but rather due to several (biochemical, enzymatic and genetic) factors that act in synergy (11).

Some research showed reduced levels of fructose in SHV, and hypothesized inadequate functioning of the seminal vesicles as the explanation (23, 24). Other studies proposed that hyperviscosity affects sperm motility which is associated with hypofunction of the seminal vesicles (7). Carpino and Siciliano studied the possible correlation between SHV and protein secretion of the epididymis, vesicles and prostate (21). They found that hyperviscosity plays no role in the semen coagulation process. Mendeluk et al. (25) reported that there is no difference in the level of total proteins, DNA, or in the percentage of water content in hyperviscous semen compared to non-viscous semen. In addition, they observed that lysozyme has no direct role in SHV, although a deficiency in cases of chronic infections could be an aggravating factor from a clinical standpoint (19, 26). Rossi et al. (27) showed that genetic factors can influence the fluidity of semen in patients with hyperviscosity.

The other interest is the possible correlation between SHV and infections or inflammation of the genital tract. There are conflicting conclusions about this area of study. According to Munuce et al. (22), there is no association between SHV, positivity in semen culture, leukospermia, or the presence of sperm antibodies. In addition, Dondero et al. (28) reported that there is no correlation between SHV and human immunodeficiency virus infection. However, Wang et al. (29) found that *Ureaplasma urealyticum* infections were associated with SHV. In a study by La Vignera et al. (18), they evaluated whether the viscosity of semen in 30 patients with male accessory gland infection is related to the extension of the inflammatory process in the various glands. Viscosity of semen sample from patients with accessory gland infection was significantly greater than that in the control groups. Elia et al. (11) also proposed that anti-inflammatory therapy can successfully treat mild SHV, in which the condition seems to be the result of infection or inflammation.

Another area of particular interest is the possible correlation between SHV and impaired semen antioxidants system that leads to an increase in oxidative stress and DNA damage. Siciliano et al. (13) have demonstrated, for the first time, a severe impairment of the high and low molecular weight antioxidant in semen of patients with hyperviscosity. They proposed that the low sperm motility is probably related to high oxidative stress in hyperviscous semen. Aydemir et al. (30) also point out increased oxidative damage as a possible risk factor for SHV. They reported that MDA levels in seminal plasma of infertile patients was significantly higher than non-viscous samples, suggesting hyperviscous semen may be related to an increase in oxidative damage in infertile men. Our results are, therefore, in compliance with these mentioned-studies. In our findings, the mean value of TAC concentration in seminal plasma of fertile (control) group was significantly higher than both infertile groups. Patients with hyperviscous semen had significantly lower mean TAC value compared with non-hyperviscous group. On the other hand, the mean of MDA levels in seminal plasma of fertile men was higher than that in both infertile patients groups.

According to our study and other researches, a severe impairment of seminal antioxidative systems and an increased sperm membrane lipid peroxidation are considered as indications for low quality of sperm in patients with hyperviscous semen. But the mechanism in which the depletion of antioxidants in the seminal plasma of patients occurs has not been fully elucidated. Furthermore one consequence of antioxidants deficiency in hyperviscous semen can be an increase in oxidative damage induced by ROS. Leukocytes and morphologically abnormal spermatozoa are the main sources of ROS in human semen (11). Patients with hyperviscous semen have high percentage of leukocytes and abnormal sperm compared with non-hyperviscous men (12, 18). Therefore, increased ROS in the seminal plasma of patients with hyperviscous semen may decrease the effective concentration of antioxidants and increase the harmful effects of ROS to sperm cells, especially lipid peroxidation that is associated with abnormal sperm parameters. The limitation of this study was the lack of ROS determination in semen of hyperviscous samples to find a good relationship between ROS levels and lipid peroxidation in viscous semen. Although high levels of ROS in semen of infertile men have been reported in previous studies, it can be investigated by our group in future study.

According to our study and other research, a severe impairment of seminal antioxidative systems and an increased sperm membrane lipid peroxidation are considered as indications for low quality of sperm in patients with hyperviscous semen. But the mechanism in which antioxidants in the seminal plasma of patients with hyperviscous semen occurs has not been fully elucidated. It can also say that treatment with antioxidants may be useful in patients showing abnormal semen consistency to protect sperm cells by peroxidative damage and to improve their functional properties which is merited future studies.
